# Prediction of T4SS Effector Proteins for *Anaplasma phagocytophilum* Using OPT4e, A New Software Tool

**DOI:** 10.3389/fmicb.2019.01391

**Published:** 2019-06-21

**Authors:** Zhila Esna Ashari, Kelly A. Brayton, Shira L. Broschat

**Affiliations:** ^1^School of Electrical Engineering and Computer Science, Washington State University, Pullman, WA, United States; ^2^Department of Veterinary Microbiology and Pathology, Washington State University, Pullman, WA, United States; ^3^Paul G. Allen School for Global Animal Health, Washington State University, Pullman, WA, United States

**Keywords:** T4SS effector proteins, machine learning, *Anaplasma phagocytophilum*, protein prediction, OPT4e software

## Abstract

Type IV secretion systems (T4SS) are used by a number of bacterial pathogens to attack the host cell. The complex protein structure of the T4SS is used to directly translocate effector proteins into host cells, often causing fatal diseases in humans and animals. Identification of effector proteins is the first step in understanding how they function to cause virulence and pathogenicity. Accurate prediction of effector proteins via a machine learning approach can assist in the process of their identification. The main goal of this study is to predict a set of candidate effectors for the tick-borne pathogen *Anaplasma phagocytophilum*, the causative agent of anaplasmosis in humans. To our knowledge, we present the first computational study for effector prediction with a focus on *A. phagocytophilum*. In a previous study, we systematically selected a set of optimal features from more than 1,000 possible protein characteristics for predicting T4SS effector candidates. This was followed by a study of the features using the proteome of *Legionella pneumophila* strain Philadelphia deduced from its complete genome. In this manuscript we introduce the OPT4e software package for Optimal-features Predictor for T4SS Effector proteins. An earlier version of OPT4e was verified using cross-validation tests, accuracy tests, and comparison with previous results for *L. pneumophila*. We use OPT4e to predict candidate effectors from the proteomes of *A. phagocytophilum* strains HZ and HGE-1 and predict 48 and 46 candidates, respectively, with 16 and 18 deemed most probable as effectors. These latter include the three known validated effectors for *A. phagocytophilum*.

## 1. Introduction

*Anaplasma phagocytophilum* is a tick-borne zoonotic Gram-negative pathogen that causes Human Granulocytic Anaplasmosis (HGA). Incidence of this potentially fatal disease is rising in the United States, with the number of cases increasing from 348 in 2000 to 5,762 in 2017 and incidence rates increasing from 1.4 cases per million people in 2000 to 17.9 cases per million in 2017. The number of cases in the United States increased 39% from 2016 to 2017 alone (CDC, 2019). Moreover, the geographic range of *A. phagocytophilum* seems to be increasing along with the range expansion of the tick vector *Ixodes scapularis* (blacklegged tick). HGA is now the third most common vector-borne infection in the United States (Dumler et al., 2005; Dumler, 2012; Bakken and Dumler, 2015; Sinclair et al., 2015; CDC, 2019).

The geographic distribution of HGA is mainly focused in the upper midwest and northeastern United States, which coincides with Lyme disease and other *I. scapularis*-transmitted diseases (CDC, 2019). The agent of Lyme disease, *Borrelia burgdorferi*, and other human pathogens such as *Babesia microti, Borrelia mayonii, Borrelia miyamotoi*, and *Ehrlichia muris eauclairensis* are also transmitted by *I. scapularis*, with co-infections with *A. phagocytophilum* reported in <10% of cases (CDC, 2019).

Some Gram-negative bacteria such as *A. phagocytophilum* have evolved specialized secretion systems, secreting proteins that interact with host cells. The type IV secretion system (T4SS) is a macromolecular complex composed of proteins that are responsible for secreting effector proteins directly into the cytosol of eukaryotic host cells. The transported proteins, called effector proteins, are instrumental agents of virulence and pathogenesis and play a key role in altering environmental niches to allow pathogen replication (Voth et al., 2010, 2012; Abby et al., 2016; Han et al., 2016), yet relatively little is known about them. A critical goal is to understand how effectors cause infection in humans and animals which requires knowledge of the function of each effector. The first step toward this goal is identifying the effectors from among the entire set of proteins in the complete genome of a bacterial pathogen with a T4SS.

In addition to experimentally validating effector proteins by means of fusion protein reporter assays in translocation studies (Voth et al., 2012; Maturana et al., 2013), a time-consuming and expensive process, several computational methods have been proposed for the prediction of effectors (Burstein et al., 2009; Yu et al., 2010; Lockwood et al., 2011; Meyer et al., 2013; Zou et al., 2013; Wang et al., 2014, 2018a,b). Accurate prediction of effector proteins greatly limits the number of proteins requiring experimental verification which reduces costs. Current computational methods use either a scoring method (Meyer et al., 2013) or a machine learning approach (Burstein et al., 2009; Zou et al., 2013; Wang et al., 2014, 2018a,b) to predict a set of candidate effectors. For example, Meyer et al. (2013) used a scoring method to predict effectors for *Legionella pneumophila* and other pathogens. Burstein et al. (2009) used machine learning to focus on the *L. pneumophila* genome while (Wang et al., 2014) studied *Helicobacter pylori* effectors. In addition, there are several reviews on T4SS effector prediction and the progress made in this area (McDermott et al., 2011; Wang et al., 2017a; An et al., 2018; Zeng and Zou, 2019) as well as several databases for curating experimentally validated effector proteins for some species (Bi et al., 2013).

The computational methods previously reported for T4SS effector prediction used different sets of protein characteristics as features for their methods. We suspect that the use of these differing feature sets explains the differences in effector predictions by the different algorithms. As a result of the disparities between the results of earlier methods, we assembled all the features used in prior studies and used a multi-level, statistical approach to determine which were the most effective in predicting effector proteins (Esna Ashari et al., 2017, 2018). Because of the number of validated effectors available for *L. pneumophila*, we then ran a number of experiments on the whole genome of *L. pneumophila* using our optimal set of features (Esna Ashari et al., 2019). A comparison of our results with the list of validated effectors and those of previous studies was highly encouraging.

Although *A. phagocytophilum* employs the T4SS to invade human cells and cause anaplasmosis, a disease sometimes fatal to humans, it has just three experimentally verified effector proteins. As such, in order to conduct further research on this increasingly important human pathogen, there is a need to identify more effector proteins. Accurate prediction of effectors will assist in this identification. In this paper we turn our attention to the prediction of effector proteins in *A. phagocytophilum*. This pathogen has not been the focus of previous computational studies for effector prediction, in part because of its lack of validated effector proteins. Because of the high accuracy of the prediction results we obtained for *L. pneumophila* using a combination of validated effectors for four different pathogens, we decided to apply our method to *A. phagocytophilum*.

In addition to applying our model for T4SS effector prediction to *A. phagocytophilum*, we also improved it based on what we learned from our previous study (Esna Ashari et al., 2019) and expanded the code to make it easy for microbiologists to use for other bacteria with T4 secretion systems. We created a software package called OPT4e, for Optimal-features Predictor for T4SS Effector proteins, that performs all the steps described in our previous studies as well as incorporating new steps, including automation of feature evaluation which is very time consuming for whole proteomes. OPT4e is specifically designed for T4SS effector protein prediction and for use on Windows, Mac OS X, and Linux operating systems. One of the main characteristics of OPT4e is that it integrates all the tools, scripts, and software needed for calculation of our optimal set of features (Esna Ashari et al., 2018) and automatically creates the set of optimal features for training or test sets. OPT4e predicts candidate effectors and groups them based on their degree of likelihood of being an effector. In addition, OPT4e can be updated to become a stronger predictor over time. Finally, OPT4e has a very simple and intuitive graphical-user interface (GUI) making it easy to use.

The remainder of the manuscript is organized as follows: First, we focus on introducing OPT4e and the steps taken to create its framework and the related algorithms. Next we explain our set of optimal features and the machine learning algorithm used for OPT4e. We then introduce the datasets used in this study for the training and test sets followed by presentation of our results. In the final section, we discuss the results we obtained for OPT4e for two input proteomes.

## 2. Materials and Methods

### 2.1. OPT4e Software

We designed and created OPT4e as a software package for the purpose of predicting effector proteins in different T4SS bacterial pathogens. OPT4e is an easy-to-use and user-friendly software package written in Python 3. Its specific features are as follows: It is based on usage of a machine learning approach for effector prediction. Each protein characteristic in a sequence is identified as a feature and is assigned the appropriate coefficient by the machine learning algorithm based on its significance as determined by the training data, and it is not necessary to determine the importance of each feature manually. Moreover, it gathers and connects multiple bioinformatic tools in order to automatically calculate and assign all the needed features and to select the best ones. Therefore, installation is simple, and there is no need to use lots of online tools or to know a specific programming language to be able to use OPT4e as is necessary for some previously developed tools (Burstein et al., 2009; Zou et al., 2013; Wang et al., 2014). In addition, OPT4e predictions are based on protein sequences and are not dependent on an entire bacterial proteome. In fact, the input to OPT4e can be a single protein sequence selected by the user. Also, OPT4e is based on predictions using a specific machine learning algorithm while taking advantage of two additional algorithms in order to present the results in three different groups of more-likely, possible, and less-likely candidate effectors. One of the most important features of OPT4e is that it can be updated over time. Thus, if a user has some new experimentally verified effectors or discovers some critical non-effectors, they can add them to the software using a few mouse clicks. The software will then include them in the training set and update the model automatically. Enriching the set of validated effectors in the software dataset will help with the accuracy of the machine learning predictions, and OPT4e will become increasingly more accurate with time.

#### 2.1.1. Framework

**Figure 1:**
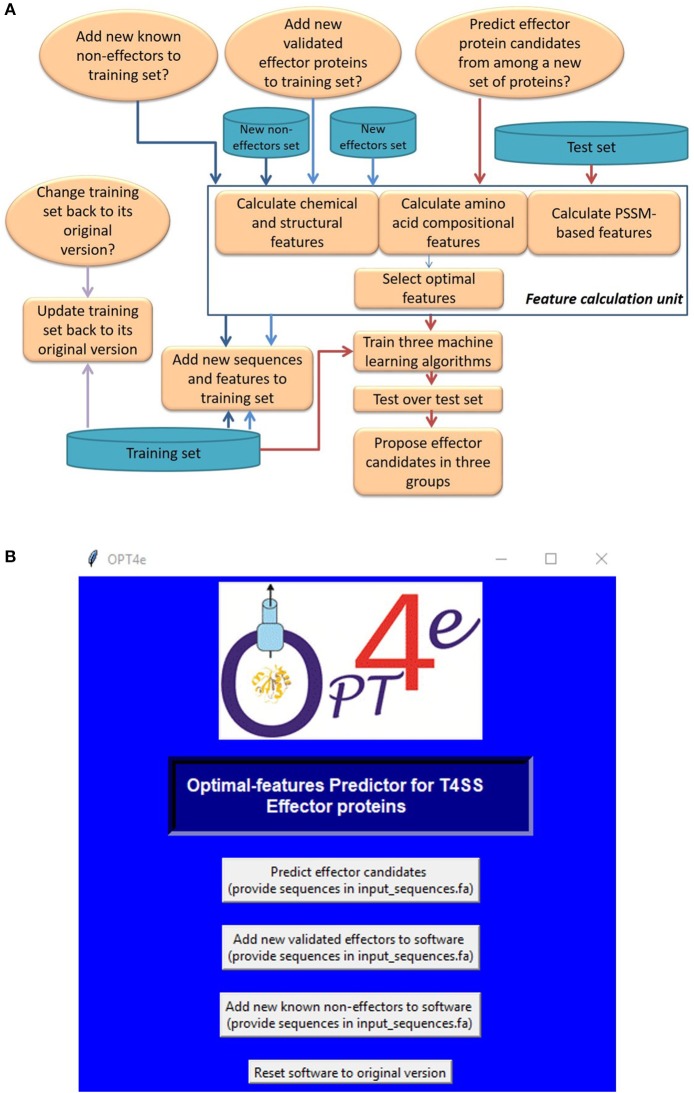
OPT4e Software: **(A)** The framework for OPT4e. **(B)** The Graphical User Interface (GUI) for OPT4e.

The framework and Graphical User Interface (GUI) for OPT4e are presented in Figure 1. First we provide an explanation of the framework, shown in Figure 1A, as follows: In the initial step a training set of known effectors and non-effectors is provided, and values for the optimal features are calculated for them automatically (Esna Ashari et al., 2018). OPT4e uses this set as its input. Note that in the first step a user has to select the appropriate button related to the purpose for using the software. If it is being used for effector prediction, the user will need to provide the test file for a protein sequence or a set of sequences in fasta-file format for classification as effectors or non-effectors by the OPT4e software. Then the software will calculate the feature values for each of the sequences provided such that they are available for machine learning prediction. The features used in this package are explained in the next section.

In the next step, OPT4e uses a support vector machine (SVM) algorithm with a radial basis function (RBF) kernel to predict effector protein sequences. This algorithm was found to give the best results as explained in Esna Ashari et al. (2019). In addition, OPT4e uses two additional classifiers (SVM with linear kernel and logistic regression) with the test sequences and uses their results to group the initially predicted effectors into three groups of more-likely (predicted by all three classifiers), possible (predicted by an additional classifier), and less-likely (predicted by just the initial SVM RBF classifier). The predicted groups of effector sequences are given as the output of the program. It should be noted that this methodology was used in the previous version of our algorithm as well (Esna Ashari et al., 2019). However, in our earlier work we used two ensemble classifiers and divided the features into three different groups for each ensemble set in addition to using the SVM with radial basis function with all the features. We found that a single classifier used with all the optimal features gave better results (Esna Ashari et al., 2019). Hence, we have replaced the ensemble classifiers with an SVM with a linear kernel and logistic regression in order to improve the model.

If a user wants to add experimentally verified effectors or some new known non-effectors to the training set to enrich it, they should select the appropriate option when using OPT4e. Then the software will automatically calculate the corresponding feature values for the new sequences and will add them to the feature set of the older training set.

We have added an option in OPT4e in case a user has made changes to the training set incorrectly or decides they do not want to change it. When the user clicks on the last button on the GUI (Figure 1B), OPT4e will reset the training data back to the original version. Finally, OPT4e is an open-source package, and users can update it as they wish.

### 2.2. Features and Feature Selection

As described in the introduction, in our earlier study we analyzed a comprehensive set of features gathered from previous computational studies performed in the field of T4SS effector protein prediction. The total number of features, including elements of vector features, was 1,027. The complete list of these features and the tools and software needed for their computation are presented in Esna Ashari et al. (2017, 2018).

We used a multi-step feature selection algorithm, described briefly in the next paragraph, to generate a set of optimal features for prediction of effector proteins consisting of 370 features. The detailed list of selected features, including the selected vector feature elements, can be found in Esna Ashari et al. (2018). The features can be grouped into chemical properties determining the way proteins interact with their environment and how effectors enter host cells (Yu et al., 2010; Zou et al., 2013), structural properties affecting protein-protein interactions between bacterial pathogens and host cells (Yu et al., 2010; Zou et al., 2013; Wang et al., 2014), compositional properties including the amino acid and dipeptide composition of protein sequences, and position-specific scoring matrix (PSSM)-related properties including PSSM composition and PSSM auto-covariance correlation composition (Zou et al., 2013; Wang et al., 2017b). The compositional properties determine the shapes and motifs of the protein sequences and, therefore, can affect the way they interact with host cells.

The first step in determining our optimal set of features was to use a filtering selection approach. For this purpose we used the *t*-test as a hypothesis testing method to filter features based on their associated *p*-values. Next we used Principal Component Analysis and Factor Analysis for dimensional reduction and to eliminate any redundancy and correlation in our feature set. The final step in our statistical approach was designing a fast backward feature selection method based on a Hosmer-Lemeshow goodness-of-fit test and using binary logistic regression. In this fashion we were able to retrieve a set of optimal features that work well together for effector prediction, and the concordance percentage from the Hosmer-Lemeshow goodness-of-fit test was still high after removal of the less related features.

### 2.3. Machine Learning Model

After selecting a set of optimal features, we designed multiple machine learning-based classifiers and tested them in order to select the most accurate predictor with our feature set (Esna Ashari et al., 2019). In due course, we focused on three classifiers. They included the SVM with the RBF kernel which is a well-known classifier and two ensemble classifiers (Esna Ashari et al., 2019). Based on 10-fold cross-validation results for our training set, results for our test set, and comparison with the results of previously developed methods, the SVM with the RBF kernel classifier was selected for further predictions, and it is the main classifier used in the OPT4e software package. As mentioned earlier, the ensemble classifiers were replaced by an SVM with a linear kernel and logistic regression.

### 2.4. Dataset

In order to create our training set, we gathered known effectors and non-effectors for four Gram-negative bacterial pathogens from the Alphaproteobacteria and Gammaproteobacteria classes. This set is composed of effectors and non-effectors from: *L. pneumophila, Coxiella burnettii, Brucella* spp., and *Bartonella* spp. Furthermore, we added the three validated effectors and multiple non-effectors from *A. phagocytophilum* to our training set. The numbers of non-effector sequences added from *A. phagocytophilum* strains HZ and HGE-1 were 115 and 120 sequences, respectively. The final training set included 1,365 sequences consisting of 432 effectors and 933 non-effectors. Moreover, we added four experimentally validated effector proteins for *Anaplasma marginale* to the training set and repeated all the experiments (Lockwood et al., 2011). Therefore, the final training set consisted of 436 effectors. The complete file of protein sequences in fasta format used in the training set is presented in Supplementary Data Sheet S1.

For this study we selected two strains of *A. phagocytophilum*, strain HZ (accession number CP000235) and strain HGE-1 (accession number APHH01000001), for use with OPT4e. These strains are composed of 1,352 and 1,148 protein sequences, respectively. We used these two sets of protein sequences as input files for OPT4e. In addition, the proteome for *L. pneumophila* strain Philadelphia with 2,942 sequences was examined. Results for the latter proteome are briefly described later to explain the performance of OPT4e. More details concerning these datasets are given in the next section.

## 3. Results

In this section we present the results obtained by OPT4e for the proteomes of *A. phagocytophilum* strain HZ and strain HGE-1. First, however, we present a brief discussion on validation of our classifier for the results obtained for the proteome of *L. pneumophila* strain Philadelphia.

### 3.1. Validation of OPT4e

We performed a thorough validation of the earlier version of our machine learning model as described in Esna Ashari et al. (2019). Briefly, in our previous study we performed 10-fold cross-validation for our training set and achieved an average accuracy of 94.05% over all folds for the SVM with radial basis function. Also, the model was verified using other performance metrics and achieved an average precision of 92.49%, an average recall of 92.00%, an average MCC (Matthews Correlation Coefficient, a measure of correlation between real and predicted values) of 0.87, and an average AUC (area under the curve) of 0.98. For further validation of our method, we tested the algorithm using the proteome for *L. pneumophila* strain Philadelphia and compared our predictions with ones from previous computational methods. Our results for effector candidates considered to be the most likely agreed with 80.5 and 72.2% of candidate effectors predicted using previous methods developed by Burstein et al. (2009) and Meyer et al. (2013), respectively. Also, the results predicted 93.7 and 99.8% of known effectors and non-effectors, respectively, from our training set (Esna Ashari et al., 2019).

As mentioned earlier, in our previous study we learned that using all the features with a single classifier gave more accurate results than separating the features and using them in an ensemble classifier (Esna Ashari et al., 2019). Thus, for OPT4e we replaced the ensemble classifiers in our model for determining more-likely, possible, and less-likely effectors. To ensure that changing to the SVM with linear kernel and logistic regression classifiers actually does give more accurate results, we used 10-fold cross validation with our *L. pneumophila* strain Philadelphia effector and non-effector proteins. We obtained accuracies of 93.73% for the SVM with linear kernel and 93.79% using logistic regression. This is in contrast to our previous ensemble results for which we obtained average accuracies of 93.64 and 92.44%.

### 3.2. Predicted Effectors for *A. phagocytophilum* Strains HZ and HGE-1

*Anaplasma phagocytophilum* strain HZ contains 1,352 protein sequences consisting of 115 known non-effectors including the protein sequences associated with the genes *rpoB* (DNA-directed RNA polymerase subunit beta), *rpoC* (DNA-directed RNA polymerase subunit beta'), and Msp2/P44. For this strain, 14 protein sequences were predicted to be more likely to be an effector protein.

*Anaplasma phagocytophilum* strain HGE-1 contains 1,148 protein sequences consisting of 120 known non-effectors including DNA pol III, delta subunit (HGE1_05467), DNA-binding protein HGE1_04712 (a helix-turn-helix DNA binding protein somewhat specific to bacteria), MerR transcriptional regulator-HGE1_05592 (a helix-turn-helix DNA binding protein somewhat specific to bacteria), type IV secretion system VirB6-HGE1_01722 (a part of the T4SS structure), putative ABC transporter, permease protein-HGE1_00015 (an outer membrane protein also found in *Escherichia coli*), thiamine biosynthesis protein ThiS-HGE1_00315 (a sulfur carrier protein common in bacterial metablolism), and Msp2/P44 sequences. For this strain, 17 protein sequences were predicted to be more likely to be an effector protein.

Table 1 lists the number of candidate effectors for both strains of *A. phagocytophilum* according to their likelihood as predicted by OPT4e.

**Table 1:** Number of effector candidate proteins for *A. phagocytophilum* strains HZ and HGE-1 before and after adding *A. marginale* validated effectors to the OPT4e training set.

	**Before adding** ***A. marginale*** **effectors**		**After adding** ***A. Marginale*** **effectors**	
	**Ap strain HZ**	**Ap strain HGE-1**	**Ap strain HZ**	**Ap strain HGE-1**
More likely	14	17	16	18
Possible	10	6	9	5
Less likely	22	23	23	23
Total	46	46	48	46

*A. phagocytophilum is indicated by Ap*.

Because *Anaplasma marginale* is more closely related to *A. phagocytophilum* than the bacteria used in our model, we added four experimentally verified effector proteins for *A. marginale* (Lockwood et al., 2011) to our training set and repeated our experiments. Two new candidate effectors were predicted for *A. phagocytophilum* strain HZ. Also, the more likely category of candidate effectors was increased by 2 and 1 for *A. phagocytophilum* strains HZ and HGE-1, respectively. Specific numbers are reported in Table 1, and all predicted candidate effectors are presented in Tables 2–**4** by locus number. In addition, Tables 2–**4** present suggestions for the order of experimental verification of candidate effectors as explained in detail in the next section.

**Table 2:** Effector candidates predicted by OPT4e.

**Suggest**	**HGE1**	**HGE1 (other)**	**HZ**	**HZ (other)**	**Notes**
	HGE1_00145		APH_0033		
*	HGE1_00220	S, T4	APH_0049	S, T4	
*	HGE1_00312	T4	APH_0068	T4	
*	HGE1_00527	S	APH_0117	S, T4	
	HGE1_00815		APH_0189		HZ: 7-aa insert at start
*	HGE1_01135	S, T4	APH_0259	S, T4	
*	HGE1_01175	T4	APH_0267	T4	HGE1: 17-aa insert at start
	HGE1_01752		APH_0382		
*	HGE1_01772		APH_0385	T4	
	HGE1_02100		APH_0385		
	HGE1_01777		APH_0386		
	HGE1_01782		APH_0387		
	HGE1_02092		APH_0452		
	HGE1_02095		APH_0453		
	HGE1_02117		APH_0453		
	HGE1_02107		APH_0455	S	Known effector
*	HGE1_02112		APH_0457	T4	
*	HGE1_02242	T4	APH_0485	T4	
*	HGE1_02492	S, T4	APH_0546	S, T4	
*	HGE1_02802	T4	APH_0633	T4	
*	HGE1_02817	T4	APH_0636	T4	HGE1: 3-aa insert at start
	HGE1_02827		APH_0641		
	HGE1_02947		APH_0670		First aa different
*	HGE1_03022		APH_0688	T4	
*	HGE1_03117		APH_0708	T4	
*	HGE1_03122	S	APH_0709	S, T4	
*	HGE1_03182	S, T4	APH_0726	S, T4	
*	HGE1_03232	S, T4	APH_0740	S, T4	Known effector
	HGE1_03297		APH_0755		
*	HGE1_03432		APH_0792	T4	
*	HGE1_03492	S	APH_0805	S, T4	
*	HGE1_03497		APH_0807	T4	
*	HGE1_03502	T4	APH_0808		
	HGE1_03532		APH_0815		
	HGE1_03557		APH_0820		
	HGE1_03697	S, T4	APH_0859	S, T4	Known effector
*	HGE1_03707	T4	APH_0861	T4	
*	HGE1_02737	T4	APH_0863	S	
*	HGE1_03892	S, T4	APH_0914	S, T4	
*	HGE1_05072	T4	APH_1167	T4	
*	HGE1_03907		APH_0916	T4	
	HGE1_03962		APH_0928		
*	HGE1_04167	T4	APH_0976	T4	
	HGE1_03507				HZ homolog not predicted
	HGE1_05977		APH_1365		First aa different
*	HGE1_05997	T4	APH_1369	T4	HZ: 6-aa insert at start
*	HGE1_06052	S	APH_1379	S	HZ: 14-aa insert in middle
*	HGE1_06067	T4	APH_1383	T4	
			APH_0239		No equivalent sequence in HGE1
			APH_0904		No equivalent sequence in HGE1
			APH_0028		No equivalent sequence in HGE1
			APH_0640		No equivalent sequence in HGE1
			APH_0816		No equivalent sequence in HGE1

*HGE1 and HZ homologs are row aligned. Blue, orange, and red text colors indicate More Likely, Possible, and Less Likely effector candidates, respectively. Black text indicates that a sequence was not predicted as an effector. The columns HGE1 (other) and HZ (other) indicate when a sequence was predicted as an effector candidate by S4TE (S) or T4EffPred (T4). The Notes column lists differences between HGE-1 and HZ homologs, and finally the Suggest column indicates effector candidates proposed for initial experimental validation based on the strength of their predictions*.

## 4. Discussion

The main goal of this study was predicting a set of candidate effectors for *A. phagocytophilum* using a new package called OPT4e which we developed for this purpose. In fact, OPT4e can be used to give reasonable candidate effector predictions for most T4SS bacteria from the Alphaproteobacteria and Gammaproteobacteria classes. For *A. phagocytophilum* strains HGE-1 and HZ, we predicted 48 and 46 candidate effectors, respectively, with 16 and 18 more likely to be effectors. All three experimentally-verified effector proteins were included in the 16 and 18 more-likely category.

We compared the differences between the predictions for the two strains and found that whenever there was a difference between the category in which an effector was predicted or an effector was not predicted for one of the strains, there was a difference between the homologous protein sequences of the two strains. These differences are noted in Table 2. In addition, five effector proteins were predicted in strain HZ for which there is no equivalent protein sequence in strain HGE-1. Strain HZ was the first *A. phagocytophilum* genome to be sequenced, and many small open reading frames (ORFs) were annotated that have not been retained in subsequent annotations (including the RefSeq for HZ). Some of these small ORFs account for the differences between the effector predictions for the two strains. Interestingly, there was one effector predicted in HGE-1 for which there was not an equivalent protein annotated in HZ. However, closer inspection of the HZ genome revealed that the sequence is present.

It should be noted that in machine learning-based prediction, an algorithm tries to fit as many training samples as it can based on the given features, and as the numbers of features and samples increase, the task increases in complexity. Also, it should be noted that the greater part of our positive training set consists of known effectors for *L. pneumophila* because it has the largest number of verified effectors. Moreover, there are only three verified effectors for *A. phagocytophilum* in our dataset. Therefore, it is possible that our set of candidate effectors for *A. phagocytophilum* include the ones that are mostly similar to *L. pneumophila* effectors. In addition, OPT4e may be detecting genes with a different signature from the rest of the genome such as those acquired by horizontal gene transfer in species where this occurs. Thus, caution is necessary when evaluating the output. It should be noted, however, that a strength of OPT4e is that it can be updated over time, and a user has the ability to add newly verified effectors to the training dataset. As a result, as new effectors for *A. phagocytophilum* are verified, they can be used in OPT4e to increase its accuracy for predicting effector proteins.

As a final note, we compared our effector candidates for *A. phagocytophilum* with those predicted by S4TE (Noroy et al., 2019) and T4EffPred (Zou et al., 2013) after we used these two programs to predict effectors for both *A. phagocytophilum* strains in our study. For HZ, OPT4e shared 13 of 48 predictions with S4TE and 27 of 92 predictions with T4EffPred. S4TE and T4EffPred shared ten predictions. Two of these were for known effectors. The third known effector was predicted by S4TE but not by T4EffPred. Thus, both OPT4e and S4TE predicted all three known effectors (see Table 2). For HGE-1, OPT4e shared 11 of 49 predictions with S4TE and 19 of 45 predictions with T4EffPred. S4TE and T4EffPred shared seven predictions. Two of these were for the homologs of known effectors. The third effector homolog was not predicted by either method; only OPT4e predicted all three.

One strategy for deciding which effector candidates to choose for experimental verification is to select from among the ones predicted by OPT4e for both strains of *A. phagocytophilum* and also predicted by one of the other two methods, S4TE or T4EffPred. There are 28 of these indicated by asterisks in Table 2, where HGE1 and HZ homologs are row aligned.

An alternative strategy and more systematic approach is to first group the predicted effectors on the basis of more-likely, probable, and less-likely and then based on predictions by the two methods, S4TE or T4effPred. Experimental verification would begin with Group 1 and proceed in order through successive groups as shown in Tables 3, 4. Table 3 is for HZ and Table 4 is for HGE-1, and for both strains Group 1 candidate effectors have literally been predicted by five different algorithms, the three from OPT4e plus S4TE and T4EffPred. For HZ, two of the three sequences in Group 1 are for known effectors, and the third known effector is in Group 2. For HGE-1, two of the sequences in Group 1 are for homologs of known effectors, and the third effector homolog is in Group 3. Thus the first three groups for each strain present excellent choices for experimental verification.

**Table 3:** Groups recommended for experimental verification of effector candidates for strain HZ.

**Effector candidates**	**Other models**	**Notes**
**Group 1**		
APH_0259	S, T4	
APH_0740	S, T4	Known effector
APH_0859	S, T4	Known effector
**Group 2**		
APH_0239	T4	
APH_0385	T4	
APH_0457	T4	
APH_0636	T4	
APH_0904	T4	
APH_0455	S	Known effector
**Group 3**		
APH_0033		
APH_0382		
APH_0385		
APH_0386		
APH_0387		
APH_0452		
APH_0453		
APH_0928		
**Group 4**		
APH_0267	T4	
APH_0633	T4	
APH_0861	T4	
APH_1167	T4	
APH_1369	T4	
APH_0863	S	
APH_1379	S	
**Group 5**		
APH_0028		
APH_0640		
APH_1365		
**Group 6**		
APH_0049	S, T4	
APH_0546	S, T4	
APH_0709	S, T4	
APH_0726	S, T4	
APH_0805	S, T4	
APH_0914	S, T4	
**Group 7**		
APH_0068	T4	
APH_0485	T4	
APH_0688	T4	
APH_0708	T4	
APH_0792	T4	
APH_0807	T4	
APH_0916	T4	
APH_0976	T4	
APH_1383	T4	
APH_0117	S	
**Group 8**		
APH_0641		
APH_0670		
APH_0755		
APH_0808		
APH_0815		
APH_0816		
APH_0820		
APH_1383		

*Groups are based on whether effector candidates are More Likely shown in blue, Probable shown in orange, and Less Likely shown in red, followed by prediction by both S4TE (S) and T4EffPred (T4), prediction by one of them, or prediction by neither. We recommend starting with Group 1 and proceeding successively through Group 8*.

**Table 4:** Groups recommended for experimental verification of effector candidates for strain HGE-1.

**Effector Candidates**	**Other Models**	**Notes**
**Group 1**		
HGE1_01135	S, T4	
HGE1_03232	S, T4	Homolog of known effector
HGE1_03697	S, T4	Homolog of known effector
**Group 2**		
HGE1_01175	T4	
HGE1_05997	T4	
**Group 3**		
HGE1_00145		
HGE1_01752		
HGE1_01772		
HGE1_01777		
HGE1_01782		
HGE1_02092		
HGE1_02095		
HGE1_02100		
HGE1_02107		Homolog of known effector
HGE1_02112		
HGE1_02117		
HGE1_03507		
HGE1_03962		
**Group 4**		
HGE1_02737	T4	
HGE1_02802	T4	
HGE1_02817	T4	
HGE1_03707	T4	
HGE1_05072	T4	
**Group 5**		
HGE1_00220	S, T4	
HGE1_02492	S, T4	
HGE1_03182	S, T4	
HGE1_03892	S, T4	
**Group 6**		
HGE1_00312	T4	
HGE1_02242	T4	
HGE1_03502	T4	
HGE1_04167	T4	
HGE1_06067	T4	
HGE1_00527	S	
HGE1_03122	S	
HGE1_03492	S	
HGE1_06052	S	
**Group 7**		
HGE1_00815		
HGE1_02827		
HGE1_03022		
HGE1_03117		
HGE1_03297		
HGE1_03432		
HGE1_03497		
HGE1_03532		
HGE1_03557		
HGE1_03907		

*Groups are based on whether effector candidates are More Likely shown in blue, Probable shown in orange, and Less Likely shown in red, followed by prediction by both S4TE (S) and T4EffPred (T4), prediction by one of them, or prediction by neither. We recommend starting with Group 1 and proceeding successively through Group 7*.

## Data Availability

The OPT4e software package as well as the datasets used for this study can be found at https://bitbucket.org/zhesna/opt4e/ and http://bcb.eecs.wsu.edu/software.

## Author Contributions

ZE developed the OPT4e software and performed dataset preparation, machine learning predictions, computational experiments, data analysis, and drafted the manuscript. SB and KB supervised, conceived, and coordinated the study and contributed to the manuscript. All authors gave final approval for publication.

### Conflict of Interest Statement

The authors declare that the research was conducted in the absence of any commercial or financial relationships that could be construed as a potential conflict of interest.
